# Comparative effectiveness of two natural interventions (drumstick leaves tea and cucumber juice) on hypertension

**DOI:** 10.6026/973206300221113

**Published:** 2026-02-28

**Authors:** Patel Daminiben Bharatbhai, Mahalakshmi B, Siva Subramanian

**Affiliations:** 1 Department of Community Health Nursing, Nootan College of Nursing, Sankalchand Patel University, Visnagar, Gujarat, India; 2 Department of Paediatric Nursing, Nootan College of Nursing, Sankalchand Patel University, Visnagar, Gujarat, India; 3 Department of Psychiatric Nursing, Nootan College of Nursing, Sankalchand Patel University, Visnagar, Gujarat, India

**Keywords:** Drumstick leaves tea, cucumber juice, *moringa oleifera*, *cucumis sativus*, hypertension, blood pressure reduction, comparative effectiveness

## Abstract

Hypertension is a major public health problem and affordable natural interventions are increasingly explored for community management. Therefore, it is of interest to compare the effectiveness of drumstick leaves tea and cucumber juice on blood pressure reduction among hypertensive patients. A comparative quasi-experimental non-equivalent pre-test-post-test design was used among 200 hypertensive patients from selected villages of Aravalli District, with 100 participants each in the drumstick leaves tea and cucumber juice groups. Both interventions produced a statistically significant reduction in systolic and diastolic blood pressure (p<0.001), with no significant difference between groups in post-test values.

## Background:

Hypertension is a major modifiable cardiovascular risk factor contributing to stroke, coronary artery disease, heart failure and chronic kidney disease globally. In rural India, the rising burden of hypertension is compounded by poor awareness, low treatment rates, suboptimal medication adherence and limited access to healthcare facilities [1]. These challenges have stimulated interest in affordable, culturally acceptable and evidence-based complementary approaches, particularly plant-derived interventions that can be easily integrated into daily dietary patterns [2]. Drumstick or *Moringa(Moringa oleifera)* is widely cultivated across India and has been used traditionally for various medicinal purposes [3]. Several studies have reported blood pressure-lowering effects of *moringa*leaf extracts and preparations, though community-based evidence using simple tea preparations remains limite [4]. Cucumber *cucumis sativus* is a commonly consumed vegetable in Indian households, valued for its cooling properties and nutritional content. Cucumber juice is rich in potassium, magnesium, dietary fiber and phytochemicals including cucurbitacins and lignans [5]. Its antihypertensive potential is attributed to high potassium content promoting sodium excretion, natural diuretic effects, antioxidant properties and possible ACE- inhibitory activity of bioactive peptides.

Despite traditional use and preliminary evidence, controlled studies evaluating cucumber juice effectiveness are scarce [6]. While individual studies have examined drumstick and cucumber separately, direct comparative evaluations of their relative effectiveness in hypertensive patients are lacking [7]. Such comparative evidence is essential for guiding clinical recommendations, patient counseling and public health interventions, particularly in resource-constrained rural settings where multiple affordable options may be available [8]. Understanding whether one intervention offers superior benefits over the other can inform evidence-based practice and optimize resource allocation [9]. Therefore, it is of interest to compare the effectiveness of drumstick leaves tea and cucumber juice on blood pressure reduction among hypertensive patients in selected villages of Aravalli District using a comparative quasi-experimental design.

## Methodology:

## Research approach and design:

A quantitative approach was employed with a comparative quasi-experimental non-equivalent group pre-test-post-test design to directly compare blood pressure outcomes between two natural interventions: drumstick leaves tea and cucumber juice.

## Setting and population:

The study was conducted in selected villages of Aravalli District, Rajasthan. The target population comprised hypertensive patients aged 21-60years residing in these communities.

## Sample and sampling technique:

A total of 200 hypertensive patients were recruited and allocated into two groups: Experimental Group I receiving drumstick leaves tea (n=100) and Experimental Group II receiving cucumber juice (n=100) using non-probability purposive sampling based on predefined inclusion and exclusion criteria. Efforts were made to ensure comparability between groups regarding age, gender, duration of hypertension and baseline blood pressure levels.

## Inclusion criteria:

[1] Adults aged 21-years with diagnosed hypertension (Stage 2: ≥140/90 mmHg)

[2] Willingness to consume assigned intervention daily for 15 days

p[3] Residing in the study area and available for follow-up

## Exclusion criteria:

[1]Pregnant or lactating women

[2] Known allergy to drumstick or cucumber

[3] Severe co-morbidities requiring hospitalization

## Data collection tools:

Data were collected using structured instruments including: (i) socio-demographic profile proforma covering age, gender, education, occupation and family income; (ii) clinical profile proforma assessing hypertension duration, family history, BMI, dietary pattern, medication type, co-morbidities and smoking/alcohol habits; and (iii) standardized blood pressure measurement record sheet. Blood pressure was measured using a calibrated aneroid sphygmomanometer following standard protocols after 5 minutes of rest in sitting position.

## Intervention protocol:

## Drumstick Leaves Tea Group (Experimental Group I):

Participants received fresh drumstick leaves (approximately 10 grams) to prepare tea by boiling in 200 ml water for 5 minutes, to be consumed once daily in the morning for 15 consecutive days, along with standard lifestyle advice.

## Cucumber Juice Group (Experimental Group II):

Participants received fresh cucumber (approximately 200 grams) to prepare juice by blending and straining, to be consumed once daily in the morning for 15 consecutive days, along with standard lifestyle advice. Blood pressure measurements were recorded at baseline (pre-test), after 7 days (post-test 1) and after 15 days (post-test 2). Adherence was monitored through daily intake logs and telephonic follow-up and any adverse effects were documented with appropriate referral.

## Data analysis:

Data were analyzed using descriptive statistics (frequency, percentage, mean and standard deviation) and inferential statistics including paired t-test for within-group changes, independent t-test for between-group comparisons, repeated measures ANOVA for time group interaction and chi-square test for associations with demographic and clinical variables. Effect size was calculated using Cohen's d. Statistical significance was set at p<0.05.

## Results:

**Figure 1:**
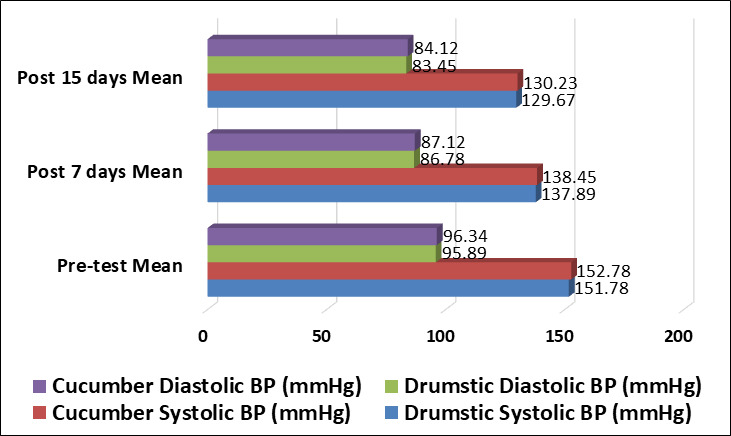
Comparison of blood pressure category distribution in Drumstick and Cucumber groups during pre-test and post-test

Table 1 show baseline characteristics were comparable in both groups, including duration of hypertension (1-2 years: drumstick 44%, cucumber 41%), family history (61% vs 64%), normal BMI (43% vs 47%), allopathic medication use (82% vs 81%), absence of co-morbidities (52% vs 50%) and no smoking/alcohol use (70% vs 72%). Table 2 and Figure 1 show that both drumstick leaves tea and cucumber juice produced a statistically significant reduction in systolic and diastolic blood pressure after 7 and 15 days of intervention. In the drumstick group, mean systolic BP decreased from 151.78±8.54 mmHg to 129.67±6.45 mmHg, while in the cucumber group it reduced from 152.12±8.89 mmHg to 130.23 ±6.78 mmHg. Similarly, diastolic BP declined from 95.89±6.34 mmHg to 83.45±5.23 mmHg in the drumstick group and from 96.34±6.56 mmHg to 84.12±5.45 mmHg in the cucumber group.

## Discussion:

This comparative study demonstrated that both drumstick leaves tea and cucumber juice are equally effective natural interventions for blood pressure reduction in hypertensive patients, with no significant difference in outcomes between the two groups. Both interventions achieved substantial and clinically meaningful reductions in systolic (approximately 22 mmHg) and diastolic (approximately 12 mmHg) blood pressure over 15 days, with progressive improvement from Stage 2 to predominantly Stage 1 hypertension or elevated categories. The comparable effectiveness observed may reflect similar underlying mechanisms of action. Drumstick leaves contain bioactive compounds such as quercetin, kaempferol and niazinin that promote nitric oxide-mediated vasodilation, reduce oxidative stress through antioxidant activity and exert anti-inflammatory effects on vascular endothelium. A study by Ghasi *et al.* [10] demonstrated that *Moringa oleifera* leaf extract significantly reduced systolic and diastolic blood pressure in experimental animal models, with mechanisms involving enhanced nitric oxide production and reduced angiotensin-converting enzyme activity. Similarly, Randriamboavonjy *et al.* [11] reported that *moringa* leaf extracts improved endothelial function and reduced oxidative stress markers in hypertensive rats, supporting our clinical findings. Cucumber juice provides high potassium content that facilitates sodium excretion, contains sterols and triterpenes with potential ACE- inhibitory activity and supplies antioxidant phytochemicals that may improve endothelial function. The finding of equivalent effectiveness has important practical implications for community-based hypertension management. Both drumstick and cucumber are widely available, affordable and culturally acceptable in rural Indian settings. Patient preference, seasonal availability, taste acceptability and ease of preparation can guide individualized recommendations without compromising clinical outcomes [12]. The magnitude of reduction observed in our study (approximately 22/12 mmHg) is clinically significant, as even modest BP reductions of 5-10 mmHg are associated with substantial decreases in cardiovascular events at population level. A meta-analysis by Ettehad *et al.* [14] demonstrated that every 10 mmHg reduction in systolic blood pressure reduces risk of major cardiovascular events by approximately 20%, stroke by 27% and heart failure by 28%, suggesting our observed reductions could translate into meaningful clinical benefits. Several strengths enhance the validity of our findings. The comparative design directly addressed the research question of relative effectiveness. Well-matched groups at baseline regarding demographic and clinical characteristics minimized confounding. Standardized intervention protocols and blood pressure measurement procedures ensured consistency. The community-based setting enhances external validity and real-world applicability [16]. However, limitations warrant consideration. The quasi-experimental design without randomization introduces potential selection bias, though baseline comparability suggests minimal impact [17]. The 15-day follow-up limits conclusions about long-term sustainability and maintenance effects. We did not include a no-intervention control group in this comparative study, limiting inferences about absolute effectiveness, though both interventions demonstrated large within-group changes [18]. Dietary sodium intake, physical activity levels and medication adherence were not systematically controlled or measured. Biochemical parameters such as serum potassium, nitric oxide metabolites, or oxidative stress markers were not assessed to elucidate mechanisms [19]. Future research should include longer follow-up periods (3-6 months) to assess sustainability, dose-response relationships, combination strategies (both interventions together) and integration with standard pharmacotherapy [13]. Randomized controlled trials with larger samples and biomarker assessments would strengthen causal inferences. Cost-effectiveness analyses comparing these interventions with pharmaceutical options would inform policy and resource allocation decisions. Arun *et al.* [15] concluded in their study that, there is remarkable reduction of blood pressure among hypertensive clients after administration of *moringa* leaves tea. Hence *moringa*leaves is found to be effective in reduction of hypertension

## Conclusion:

Drumstick leaves tea and cucumber juice showed comparable effectiveness in reducing blood pressure, with most patients shifting from Stage 2 to Stage 1 hypertension within 15 days. Both interventions can be recommended based on patient preference, availability, and cultural acceptability. These low-cost, plant-based options can support routine hypertension management in community settings.

## Figures and Tables

**Table 1:** Distribution of sample according to clinical variables (N=200)

**Clinical Variable**	**Category**	**Drumstick Group (n=100) f**	**%**	**Cucumber Group (n=100) f**	**%**
HTN Duration	1-2 years	44	44	41	41
	3-4 years	30	30	35	35
	≥ 5 years	26	26	24	24
Family History of HTN	Yes	61	61	64	64
	No	39	39	36	36
BMI Category	Underweight (≤18.5)	7	7	6	6
	Normal (18.6-24.9)	43	43	47	47
	Overweight (25-29.9)	35	35	33	33
	Obese (≥30)	15	15	14	14
Diet Pattern	Vegetarian	42	42	40	40
	Non-vegetarian	31	31	34	34
	Mixed	27	27	26	26
Type of Medications	Allopathic	82	82	81	81
	Homeopathic	9	9	10	10
	Naturopathic	3	3	3	3
	Other	6	6	6	6
Co-morbidities	None	52	52	50	50
	Metabolic	24	24	27	27
	Respiratory	11	11	12	12
	Cardiovascular	8	8	6	6
	Other	5	5	5	5
Smoking/Alcohol	Yes	30	30	28	28
	No	70	70	72	72

**Table 2:** Comparative effectiveness of drumstick leaves tea vs cucumber juice on blood pressure (N=200)

**Parameter**	**Group**	**Pre-test Mean±SD**	**Post 7 days Mean±SD**	**Post 15 days Mean±SD**	**Mean Difference**	**Paired t-test**	**p-value**
Systolic BP (mmHg)	Drumstick (n=100)	151.78±8.54	137.89±7.23	129.67±6.45	22.11	17.89	<0.001***
	Cucumber (n=100)	152.12±8.89	138.45±7.56	130.23±6.78	21.89	18.12	<0.001***
Diastolic BP (mmHg)	Drumstick (n=100)	95.89±6.34	86.78±5.67	83.45±5.23	12.44	21.45	<0.001***
	Cucumber (n=100)	96.34±6.56	87.12±5.89	84.12±5.45	12.22	21.89	<0.001***
*p<0.001 = highly significant;
NS = Not significant
